# Umbilical cord blood therapy to prevent progression of COVID-19 related pneumonia: a structured summary of a study protocol for a pilot randomised controlled trial

**DOI:** 10.1186/s13063-020-04387-y

**Published:** 2020-06-04

**Authors:** Atul Malhotra, David Ernest, Benjamin A. Rogers, David Haylock, Ashalyn Watt, Guy Moeneclaey, Graham Jenkin

**Affiliations:** 1 Monash Children’s Hospital, Melbourne, Australia; 2 Monash University Faculty of Medicine Nursing and Health Sciences, Melbourne, Australia; 3 Kabeth Consulting, Melbourne, Australia; 4 Hudson Institute of Medical Research, Melbourne, Australia

**Keywords:** COVID-19, Randomised controlled trial, Protocol, Cord blood, Stem cells, Pneumonia, ARDS

## Abstract

**Objectives:**

Objective: To undertake a pilot, feasibility RCT of umbilical cord blood derived cell therapy for treatment of adult patients infected with SARS-CoV-2 virus related moderate-to-severe pneumonia to prevent progression to severe ARDS.

Hypothesis: Expanded cord blood derived cell therapy will be feasible, well tolerated and show potential efficacy in the treatment of acute COVID-19 related moderate to severe pneumonia in adult patients because of their powerful anti-inflammatory and immunomodulatory properties.

**Trial design:**

Pilot, parallel design randomised controlled trial.

**Participants:**

The trial will recruit 24 hospitalised patients with confirmed SARS-CoV-2 infection and pneumonia from July to December 2020 at Monash Medical Centre in Melbourne, Australia.

**Intervention and comparator:**

Intervention: Intravenous injection of expanded umbilical cord blood cells at a dose of 5 million cells/kg (maximum dose - 500 million cells). Cell infusion will occur over 30-60 minutes through a peripheral intravenous cannula. Standard supportive care will continue as needed.

Comparator: Standard supportive care.

**Main outcomes:**

Safety and tolerability of cell administration within first 24 hours of administration; clinical improvement on a seven-category clinical improvement ordinal scale.

**Randomisation:**

Randomisation will be done using computer generated allocation to intervention/ control groups in a 1:1 ratio (in blocks of 6) using sealed opaque envelopes.

**Blinding (masking):**

This will be an unblinded study, given that it is the first study using expanded cord blood cells in COVID-19 patients. There will be no placebo infusion.

**Numbers to be randomised (sample size):**

Twelve participants in each group. Total *n*=24.

**Trial Status:**

CBC-19 protocol v2, dated 23^rd^ April 2020. Recruitment has not started yet. Estimated recruitment timeline is between 1st July – 31st December 2020.

**Trial registration:**

Australian New Zealand Clinical Trials Registry, ACTRN12620000478910, registered 16th April 2020.

**Full protocol:**

The full protocol is attached as an additional file, accessible from the Trials website (Additional file 1). In the interest in expediting dissemination of this material, the familiar formatting has been eliminated; this Letter serves as a summary of the key elements of the full protocol.

## Supplementary information

**Additional file 1.** CBC-19 Research Protocol v2.

## Data Availability

Not applicable.

