# Supplementary material for: Umbilical cord blood therapy to prevent progression of COVID-19 related pneumonia: a structured summary of a study protocol for a pilot randomised controlled trial
Source: Trials. 2020 Jun 4;21:474. doi: 10.1186/s13063-020-04387-y (PMC7269684; doi:10.1186/s13063-020-04387-y)
Supplement: Supplementary file 1 — Additional file 1. CBC-19 Research Protocol v2. [file 13063_2020_4387_MOESM1_ESM.docx]

**CBC-19 Research Protocol v2**

**Title:**

**C**ord **B**lood Therapy to prevent progression of **C**OVID**-19** related pneumonia

**Short Title:**

CBC-19 study

**Type of study:**

Pilot RCT, Investigator-led

**Trial Site:**

Monash Medical Centre

246 Clayton Rd

Clayton VIC 3168

**Trial Laboratory:**

Monash Health Translation Precinct Cell Therapies Platform

Translation Research Facility

Clayton VIC 3168

**Industry Partner:**

Cell Care Australia

Heatherton VIC 3202

**Coordinating Principal Investigator:**

Dr Atul Malhotra

Monash Children’s Hospital

246 Clayton Road

Clayton VIC 3168

Tel: +61 3 8752 3650

Fax: +61 3 8572 3649

E-mail: atul.malhotra@monash.edu

**Trial statement:** This study will be conducted according to the protocol and will conform to good clinical practice and all applicable regulatory requirements.

**Introduction:**

**The Problem:** As of 23^rd^ April 2020, there have been over 3 million confirmed cases of SARS-CoV-2 infections resulting in more than 200,000 deaths worldwide (1), most commonly due to severe pneumonia causing multi-organ failure (2). Whilst, to date, there have been 81 deaths in Australia amongst around 6,000 COVID-19 diagnosed positive patients, this number is anticipated to continue to increase in the next few weeks (1). Patients infected with SARS-CoV-2 virus present with varying degrees of symptoms, including mild to moderate symptoms (81%), the disease state called COVID-19 (14%) similar to a cold or flu and, in severe cases, 5-6% of confirmed patients progress to lower respiratory tract infection, severe pneumonia, respiratory failure, shock and multi organ dysfunction, including ARDS (2). A recent WHO analysis has confirmed that many patients have become critically ill with severe pneumonia and that this is the primary cause of death for these patients (3). Currently, approximately 1% of confirmed cases in Australia will die. Although the spread of the virus is being mitigated to some extent in Australia, infectious disease clinicians advise that the pandemic will remain active for several months to come, while a “second wave” of infections is anticipated once restrictions on movement are relaxed.

**Background:** Almost all serious consequences of COVID-19 feature pneumonia. When SARS-CoV-2 enters the lungs, it triggers the body’s immune response to attack the virus, resulting in localised inflammation. Increasing evidence suggests that a subgroup of these patients, with severe COVID-19, may develop ‘**cytokine storm syndrome’**, an overreaction of the body’s immune system to the virus caused by localised production of inflammatory factors (cytokines) by infiltration of inflammatory cells. The localised inflammatory response can result in hyper-inflammation causing serious harm to affected organs resulting in multi-organ failure and, if untreated, the cytokine storm syndrome, which is usually fatal (4).

**The Need:** There is a major unmet need for a safe and effective treatment for COVID-19 infected patients, especially in moderate to severe cases of pneumonia. Avoiding the “cytokine storm” may be a key factor for the treatment of severe COVID-19 infected patients. A range of techniques are being investigated to mitigate the damage caused by moderate to severe pneumonia due to SARS-CoV-2 (4), before it becomes life threatening. Many are still in the early stages of development and there are, thus, currently no proven therapies for COVID-19 related severe pneumonia; meaning the management of the condition has been primarily related to organ failure support, including mechanical ventilation for worsening respiratory failure (4). A more general approach, targeting the hyper-inflammatory reaction, along with immunomodulation of an over reactive immune system, rather than use of either anti-virals or specific cytokine inhibitors or anti-inflammatory medications alone may provide greater benefit to these patients. **Umbilical Cord Blood (UCB) derived cell therapy with proven anti-inflammatory and immunoregulatory properties may provide such a therapeutic option.**

**Rationale:**

**Umbilical Cord Blood derived cells:** UCB is a rich source of Haematopoietic Stem Cells (HSCs) that have been used in clinical therapy (transplantation) to treat haematological disorders for over 30 years (5) and, in Australia, over 500 UCB units have been released to treat a variety of approved conditions (6). Although UCB has been used primarily, as a source of haemopoietic stem cells, more recent evidence suggests that it is a therapeutically important source of other cell populations with **anti-inflammatory, immune-regulatory and vasculogenic properties**. These include not only mesenchymal stromal cells (MSCs) but also endothelial progenitor cells and immature immune cells, including potent Myeloid-Derived Suppressor Cells (MDSCs) and T-Regulatory cells (Tregs) both of which can be readily expanded from UCB (7, 8). There are currently over 60 registered clinical trials underway worldwide investigating the potential of UCB cells to act as a regenerative treatment for an expanding and diverse list of medical conditions (9), with multiple units already being released from private cord blood banks for regenerative medicine applications (10). This includes releases from Cell Care (a private cord blood bank) for use in Australia’s first clinical trial of cord blood infusion as a possible treatment for cerebral palsy and of Type 1 Diabetes (9).

Several very recent studies have reported the use of MSCs, derived from multiple sources, in COVID-19 applications (11, 12) and clinical trials of Umbilical Cord derived MSCs are planned (clinicaltrials.gov, “COVID-19” & “MSC” & “lung”). We believe that **UCB derived cell administration will value add to the potential efficacy observed with MSCs alone**, particularly during the moderate to severe phase of the disease. Unlike adult tissue sources of MSCs like adipose tissue and bone marrow, in which MDSCs and Tregs are barely detectable, significant proportion of these immunomodulatory cells are found in umbilical cord blood where they balance fetal immune responses and prevent aggressive inflammatory responses (13). The MDSC population represents an innate immune cell subset, composed of a heterogeneous mix of early myeloid progenitors, immature granulocytes, type-2 macrophages, pre-mast cells and immature dendritic cells at different stages of differentiation and characterised by its capacity to suppress T cell immunity. Besides being an excellent source of immune cells for controlling immune-regulated destruction of endogenous cells, such as that observed in prevention of destruction of beta cells in type 1 diabetes (9), UCB cells provide a treatment modality to address inflammatory reactions in the brain as observed in cerebral palsy, autism and following acute brain injury (5). Preclinical studies are now emerging on the use of UCB in the treatment of respiratory conditions (see below) and there are currently 22 trials listed in “ClinicalTrials.gov” for use of umbilical cord blood in respiratory conditions in neonates and adults (“Respiratory” & “Umbilical Cord Blood”).

**Pre-clinical and clinical studies:** We have used animal models of preterm and perinatal conditions to show that UCB derived cell administration is protective, as well as facilitating tissue repair and regeneration following injury. We have demonstrated, in multiple studies, that these effects are mediated via their **anti-inflammatory, immuno-modulatory, anti-apoptotic, and growth factor support**, rather than by direct cell replacement. We have also shown that these cells protect vasculature and stimulate vasculogenesis by release of vasogenic factors (14). Specifically, with respect to the lung, we have shown that UCB treatment in ventilated lambs increased angiogenesis, as evidenced by increased Vascular Endothelial Growth Factor expression and vessel density, attenuated immune cell infiltration into lungs and modulated the injurious effect of Ventilation Induced Lung Injury (15). In addition, we have demonstrated that administration of UCB derived cells could have beneficial effects by **preventing or attenuating the cytokine storm** since we have shown that these cells down-regulate TNF-alpha, a key inflammatory cytokine, release following a lipopolysaccharide (LPS) induced inflammatory challenge (16). Further studies in murine neonatal and adult models of have reported on the effects of human UCB derived cells on respiratory physiology i.e. reduction of central airway resistance to normoxic levels and improvement of alveolarisation, lung compliance and elastance (17, 18). Treatment also inhibited lung vascular injury, evident by decreased lung vascular permeability, attenuation of expression of pro-inflammatory mediators and, importantly with respect to this proposal, lung inflammation was returned to normal levels after an inflammatory LPS challenge, causing a marked increase of survival rate (18). **Taken together, these preclinical data clearly demonstrate the protective effects of UCB derived cells on acute lung injury.**

UCB cells have been widely used in multiple non-haematopoietic cell-based therapies. Our group has been directly involved in the first Phase 1 Trial of the use of UCB in treatment of confirmed cerebral palsy in children in Australia (SCUBI Trial (19) and, recently, we obtained ethics approval to administer autologous UCB cells to extremely preterm neonates in a Phase 1 Safety and Feasibility Trial (CORD SaFe Trial, ACTRN12619001637134) using our Cell Therapy Platform, at the Monash Health Translation Precinct, MHTP, to prepare UCB cells released from Cell Care for clinical use. It has been demonstrated that, after intravenous infusion, the majority of UCB derived cells accumulate in the lung, the “pulmonary first passage effect”, which is also the major site of SARS-CoV-2 virus entry and consequent lung inflammation (20, 21). In addition, exciting new evidence, in several recent reports, demonstrate that the first step of COVID-19 pathogenesis is that the virus specifically recognises the angiotensin I converting enzyme 2 receptor (ACE2) by its spike protein. Spike protein priming is also essential for the host cell entry and spread. ACE2 is a co-receptor for viral entry for SARS-CoV-2; and ACE2 has a broad expression pattern in the human body, with strong expression in type II alveolar cells in the lungs (22). The lung is, thus, an obvious choice for therapeutic intervention in COVID-19. **Since UCB derived cells have immunomodulatory, in addition to anti-inflammatory, effects, localisation of reparative UCB cells to the lung should improve the pulmonary microenvironment, protect alveolar epithelial cells, prevent pulmonary fibrosis and improve lung function**.

**Expansion of UCB cells:** While we are confident that UCB cells will result in improvement of pulmonary function in COVID-19 infections, we are concerned that we will be able to source sufficient unexpanded UCB units to treat severe cases of COVID-19 infections in the immediate short term. Consequently, cord blood science in general, and our lab in particular, has focused on the ability to expand the number of cells in cord blood to boost utility in both current and future medical uses, i.e. in transplantation, in immune therapy and in regenerative medicine. In collaboration with Cell Care Australia, we have developed (23), modified and undertaken in vitro and in vivo preclinical studies of expanded UCB derived cells that reliably and reproducibly results of not only a higher number of haematopoietic stem cells but also progenitor cells, T-cell precursors and MDSCs (including pre-dendritic and pre-mast cells) that display highly potent immune-regulatory capacities. These observations are in line with the observations made during clinical trials of expanded cord blood transplantation for haematological malignancies (24, 25).

Expanded UCB derived cells have already been shown to attenuate inflammatory lung injury (18) and the expansion technology has received Orphan Drug Designation (FDA, Dec 2018) in the US. Using commercially available systems, it permits expansion of cord blood units 100-fold within 7 days and over 1,000-fold following a further 7-day incubation. This would provide ample expanded cells for our trial and could permit production of sufficient cells for multiple dosing of patients in the future. Expanded UCB cells are currently undergoing Phase 2 trials in the US and Canada (24) and results of a Phase I/II trial of cord blood transplantation of expanded UCB cells have recently been published (25). These observations underline the potential benefits attributed to the immune-regulatory cells in the expanded cell population, as demonstrated by the reduction of the risk to severe acute and chronic GVHD (25).

**Methods:**

**Aims:** The objective of this proposal is to undertake a pilot, feasibility RCT of a cell-based therapy for treatment of patients infected with SARS-CoV-2 virus related pneumonia to prevent progression to severe ARDS**.** The goal is to roll out an adequately powered, double-blind, randomised controlled trial after this pilot RCT.

**Hypothesis:** Expanded UCB derived cell therapy will be feasible, well tolerated and show potential efficacy in the treatment of acute COVID-19 related moderate to severe pneumonia as a result of their powerful anti-inflammatory and immunomodulatory properties.

**Collection and Expansion of UCB cells:** UCB is already being collected (from healthy pregnant volunteers at term gestation) and processed under strict TGA guidelines. The frozen UCB units will be sourced from Cell Care’s cord blood banks and released to our PC2 and certified grade D, ISO 9001 accredited Cell Therapy Platform Facility at the Monash Health Translation Precinct where they will be thawed, expanded using our expansion system and prepared for IV administration to eligible consented patients.

Once expanded, the cells will be fully tested, for microbes and cell viability, aliquoted and frozen using our GMP compliant, standard operating procedure (SOP) for cell expansion that is being developed. Prior to infusion, the requisite number of cells will be thawed and prepared for infusion. All SOPs for collection, processing, characterisation, storage, release, and preparation for infusion are currently in place for use of UCB cells for another current UCB trial that we are undertaking (CORD SaFe Trial, ACTRN12619001637134).

*Study design*

Pilot, safety and feasibility, randomised controlled trial.

*Blinding and Randomisation*

This will be an unblinded study, given that it is the first of its kind study (using expanded UCB cells) in COVID-19 patients. There will be no placebo infusion. Randomisation will be done by lead PI using computer generated allocation to intervention/ control groups (in blocks of 6 patients) by using sealed opaque envelopes prepared by an independent research nurse.

***P****opulation*

*Inclusion Criteria*:

Hospitalised patients with confirmed SARS-CoV2 infection (based on appropriate nucleic acid or other testing) meeting the following three criteria:

1. Expected to remain an in-patient for at least 48 hours,
2. Features consistent with viral pneumonia on Chest CT or X-Ray (in the opinion of the investigator), and
3. Are unable to maintain a peripheral oxygen saturation of 95% or greater in room air

*Exclusion Criteria:*

1. Patients with known allergies to stem cell preparations.
2. Patients with known significant lung disease before COVID-19 presentation.
3. Patients receiving invasive/ mechanical ventilatory support.
4. Patients with underlying disease, comorbidities or clinical status that, in the opinion of the investigator, suggests death is imminent and/or likely to occur in the next 48 hours.
5. Patients currently on other investigational cell therapies.
6. Patients with known pregnancy.
7. Patient unable to provide consent.
8. The treating team deems that enrolment in the study is not in the best interests of the patient.

***I****ntervention group (n=12)*

Intravenous injection of expanded UCB cells at a dose of 5 million cells/kg. Maximum dose of 500 million expanded UCB cells. Standard supportive care will continue as needed.

The cell number/dosage should be easily achievable with the expansion technology that we have developed and reflects cell numbers currently being used in other COVID-19 MSC trials (12). Cell infusion will occur over 30-60 minutes through a peripheral intravenous cannula.

***C****omparison group (n=12)*

Standard supportive care will continue as needed*.*

***O****utcomes*

*Primary Outcomes:* Safety and tolerability of UCB cell administration (absence of immediate and short-term negative side effects); clinical improvement on a seven-category ordinal scale.

The seven-category ordinal scale consists of: 1, not hospitalised with resumption of normal activities; 2, not hospitalised, but unable to resume normal activities; 3, hospitalised, not requiring supplemental oxygen; 4, hospitalised, requiring supplemental oxygen; 5, hospitalised, requiring humidified nasal high-flow oxygen therapy, non-invasive mechanical ventilation, or both; 6, hospitalised, requiring invasive mechanical ventilation, ECMO or both; and 7, death. This ordinal scale has been used in previous Influenza and some COVID-19 trials (26, 27).

*Secondary Outcomes:* Time to clinical stability (defined as the time from study randomisation until resolution of symptoms leading to discharge from hospital), individual symptom resolution, chest imaging changes (if available), need and duration of mechanical ventilation, incidence of secondary culture positive bacterial infections, and all-cause mortality (30- and 90- day). Immune response will also be studied using assay of inflammatory factors (IL-1b, IL-6, TNF-α and IL-10) from blood samples obtained prior to UCB cell infusion and at 1- and 7-days post infusion.

***T****imeframe*

6-12 months.

*Sample size and statistics*

As this is a pilot, feasibility RCT, a convenience sample size of 12 in each arm has been decided. Further, given the pilot nature of this RCT, significant efficacy is not expected, and hence only descriptive and interpretive statistics will be performed to compare groups as appropriate. This trial will help design an appropriately powered larger trial if treatment is well tolerated.

*Monitoring*

Regular vital monitoring for 24 hours post cell infusion will be conducted as detailed in the case report form.

Daily clinical evaluation of symptoms and classification according to the 7-category ordinal scale until one of the following time points: a) resolution of clinical symptoms b) death. In the hospital, this monitoring will be performed by review of the Electronic Medical Record and discussion with the treating team (unless an investigator is a member of the treating team and has direct contact with the patient). In the community, this will be performed by regular telephone contact. Follow up for other morbidities with 30- and 90-day telephonic contact will also be undertaken.

Routine blood tests, including full blood examination, C-Reactive Protein, liver and renal function, coagulation profile will be done as per treating clinical team directions.

Blood tests for immune response will be done before, and 24 hrs and 7 days after UCB cell infusion.

*Safety Reporting*

*Categorising safety related events:* Events will be categorised as per the National Health and Medical Research Council 2016 recommendation:

**Adverse event (AE)** Any untoward medical occurrence in a patient or clinical trial participant administered a medicinal/ biological product and that does not necessarily have a causal relationship with this treatment.

**Adverse Reaction (AR)** Any untoward and unintended response to an investigational medicinal/ biological product related to any dose administered.

**Serious AE/AR (SAE/SAR)** Any adverse event/adverse reaction that results in death, is life-threatening, requires hospitalisation or prolongation of existing hospitalisation, results in persistent or significant disability or incapacity.

**Suspected Unexpected Serious Adverse Reaction (SUSAR)** An adverse reaction that is both serious and unexpected.

*Data Safety Monitoring Board (DSMB)*

An independent DSMB is being formed comprising of an intensivist, an infectious disease physician, and a scientist familiar with cell-based therapies to review all AE/ AR/ SAE/ SAR/ SUSARs and pre-decided reporting times (interim and final). Adverse incidents will be reported to the DSMB within 1 week of occurrence.

*Reporting to HREC*

The following reports will be submitted to HREC:

1. Any adverse event/ reaction deemed suitable by DSMB to temporarily halt the trial pending review.
2. An interim report after recruitment of 12 patients.
3. Any updates to protocol/ PICF.

*Data management*

Data will be collected prospectively by trial investigators and sourced from bedside clinical charts, monitors, electronic medical records and direct patient feedback. Data will be collected on a paper CRF and recorded in an electronic database. Paper records will be stored in a locked filing cabinet and electronic records will be kept on password protected documents. Data will be stored for 15 years.

*Ethics and oversight*

The clinical trial will be conducted in compliance with the protocol approved by Monash Health HREC and with the NHMRC National Statement on Ethical conduct in Human Research 2007 (updated May 2015). Fully informed, written consent will be obtained as per the participant information and consent form. Participation will be voluntary and option of withdrawal from trial will be possible at any stage. The trial will be overseen by investigator team members drawn from our ICU and infectious diseases teams, while the lead PI will have overall trial oversight and interaction with the DSMB and HREC.

**Expected milestones and Key Performance Indicators:**

*Preparation for expansion of UCB cells* has already commenced and sufficient cells for first infusion will be ready by June 2020.

*Recruitment* of 24 patients to this pilot feasibility RCT, will be completed within 6-12 months of commencement.

*First participant* recruitment will commence in June 2020, subject to all SOPs being validated. We envisage recruitment of at least 4-6 patients, treatment and control, per month thereafter.

*Reporting* to our DSMB and HREC will be made at least twice (after recruitment of 12 patients and at completion), or following any adverse outcomes associated with the trial. Final report and preparation of manuscript for publication within 12 months of commencement of trial.

**References**

1. Coronavirus Resource Center 2020 [Available from: <https://coronavirus.jhu.edu/map.html>.

2. Chen N, Zhou M, Dong X, Qu J, Gong F, Han Y, et al. Epidemiological and clinical characteristics of 99 cases of 2019 novel coronavirus pneumonia in Wuhan, China: a descriptive study. Lancet (London, England). 2020;395(10223):507-13.

3. Coronavirus disease (COVID-19): Uptodate; 2020 [Available from: <https://www.uptodate.com/contents/coronavirus-disease-2019>.

4. Mehta P, McAuley DF, Brown M, Sanchez E, Tattersall RS, Manson JJ. COVID-19: consider cytokine storm syndromes and immunosuppression. Lancet (London, England). 2020;395(10229):1033-4.

5. Ballen K. Update on umbilical cord blood transplantation. F1000Research. 2017;6:1556.

6. About Cord Blood [Available from: <https://www.cellcare.com.au/about-cord-banking/about-cord-blood/>.

7. Kolahian S, Oz HH, Zhou B, Griessinger CM, Rieber N, Hartl D. The emerging role of myeloid-derived suppressor cells in lung diseases. The European respiratory journal. 2016;47(3):967-77.

8. Seay HR, Putnam AL, Cserny J, Posgai AL, Rosenau EH, Wingard JR, et al. Expansion of Human Tregs from Cryopreserved Umbilical Cord Blood for GMP-Compliant Autologous Adoptive Cell Transfer Therapy. Molecular therapy Methods & clinical development. 2017;4:178-91.

9. Cord blood and tissue research and trials [Available from: <https://www.cellcare.com.au/research-and-trials/cord-blood-and-tissue-research-and-trials/>.

10. Ballen KK, Verter F, Kurtzberg J. Umbilical cord blood donation: public or private? Bone marrow transplantation. 2015;50(10):1271-8.

11. Chen J, Hu C, Chen L, Tang L, Zhu Y, Xu X, et al. Clinical Study of Mesenchymal Stem Cell Treatment for Acute Respiratory Distress Syndrome Induced by Epidemic Influenza A (H7N9) Infection: A Hint for COVID-19 Treatment. Engineering. 2020.

12. Shetty AK. Mesenchymal Stem Cell Infusion Shows Promise for Combating Coronavirus (COVID-19)- Induced Pneumonia. Aging and disease. 2020;11(2):462-4.

13. Ahmadi M, Mohammadi M, Ali-Hassanzadeh M, Zare M, Gharesi-Fard B. MDSCs in pregnancy: Critical players for a balanced immune system at the feto-maternal interface. Cellular immunology. 2019;346:103990.

14. McDonald CA, Penny TR, Paton MCB, Sutherland AE, Nekkanti L, Yawno T, et al. Effects of umbilical cord blood cells, and subtypes, to reduce neuroinflammation following perinatal hypoxic-ischemic brain injury. Journal of neuroinflammation. 2018;15(1):47.

15. Allison BJ, Youn H, Malhotra A, McDonald CA, Castillo-Melendez M, Pham Y, et al. Is Umbilical Cord Blood Therapy an Effective Treatment for Early Lung Injury in Growth Restriction? Frontiers in endocrinology. 2020;11:86.

16. Li J, Yawno T, Sutherland A, Loose J, Nitsos I, Bischof R, et al. Preterm white matter brain injury is prevented by early administration of umbilical cord blood cells. Experimental neurology. 2016;283(Pt A):179-87.

17. Mills DR, Mao Q, Chu S, Falcon Girard K, Kraus M, Padbury JF, et al. Effects of human umbilical cord blood mononuclear cells on respiratory system mechanics in a murine model of neonatal lung injury. Experimental lung research. 2017;43(2):66-81.

18. Huang X, Sun K, Zhao YD, Vogel SM, Song Y, Mahmud N, et al. Human CD34+ progenitor cells freshly isolated from umbilical cord blood attenuate inflammatory lung injury following LPS challenge. PloS one. 2014;9(2):e88814.

19. Crompton K, Novak I, Fahey M, Badawi N, Wallace E, Lee K, et al. Single group multisite safety trial of sibling cord blood cell infusion to children with cerebral palsy: study protocol and rationale. BMJ open. 2020;10(3):e034974.

20. Galieva LR, Mukhamedshina YO, Arkhipova SS, Rizvanov AA. Human Umbilical Cord Blood Cell Transplantation in Neuroregenerative Strategies. Frontiers in pharmacology. 2017;8:628.

21. Medina E, Hartl D. Myeloid-Derived Suppressor Cells in Infection: A General Overview. Journal of innate immunity. 2018;10(5-6):407-13.

22. Patel AB, Verma A. COVID-19 and Angiotensin-Converting Enzyme Inhibitors and Angiotensin Receptor Blockers: What Is the Evidence? JAMA. 2020.

23. Tiwari A, Tursky ML, Mushahary D, Wasnik S, Collier FM, Suma K, et al. Ex vivo expansion of haematopoietic stem/progenitor cells from human umbilical cord blood on acellular scaffolds prepared from MS-5 stromal cell line. Journal of tissue engineering and regenerative medicine. 2013;7(11):871-83.

24. Clinical Trials [Available from: <https://excellthera.com/clinical-trials/>.

25. Cohen S, Roy J, Lachance S, Delisle JS, Marinier A, Busque L, et al. Hematopoietic stem cell transplantation using single UM171-expanded cord blood: a single-arm, phase 1-2 safety and feasibility study. The Lancet Haematology. 2020;7(2):e134-e45.

26. Wang Y, Fan G, Salam A, Horby P, Hayden FG, Chen C, et al. Comparative effectiveness of combined favipiravir and oseltamivir therapy versus oseltamivir monotherapy in critically ill patients with influenza virus infection. The Journal of infectious diseases. 2019.

27. Cao B, Wang Y, Wen D, Liu W, Wang J, Fan G, et al. A Trial of Lopinavir-Ritonavir in Adults Hospitalized with Severe Covid-19. The New England journal of medicine. 2020.
